# Oligo-FISH barcode chromosome identification system provides novel insights into the natural chromosome aberrations propensity in the autotetraploid cultivated alfalfa

**DOI:** 10.1093/hr/uhae266

**Published:** 2024-09-20

**Authors:** Zhuang Meng, Qian Zheng, Wei Wang, Yuanbin Zhu, Yuanhao Li, Fulin Dong, Wenjun Luo, Zhiliang Zhang, Fei Wang, Haitao Shen, Quanliang Xie, Hongbin Li

**Affiliations:** Key Laboratory of Xinjiang Phytomedicine Resource and Utilization of Ministry of Education, Key Laboratory of Oasis Town and Mountain-basin System Ecology of Xinjiang Production and Construction Corps, College of Life Sciences, Shihezi University, Shihezi 832003, China; Key Laboratory of Xinjiang Phytomedicine Resource and Utilization of Ministry of Education, Key Laboratory of Oasis Town and Mountain-basin System Ecology of Xinjiang Production and Construction Corps, College of Life Sciences, Shihezi University, Shihezi 832003, China; Key Laboratory of Xinjiang Phytomedicine Resource and Utilization of Ministry of Education, Key Laboratory of Oasis Town and Mountain-basin System Ecology of Xinjiang Production and Construction Corps, College of Life Sciences, Shihezi University, Shihezi 832003, China; College of Life Sciences, Northwest A&F University, Yangling, Shaanxi, China; Key Laboratory of Xinjiang Phytomedicine Resource and Utilization of Ministry of Education, Key Laboratory of Oasis Town and Mountain-basin System Ecology of Xinjiang Production and Construction Corps, College of Life Sciences, Shihezi University, Shihezi 832003, China; Key Laboratory of Xinjiang Phytomedicine Resource and Utilization of Ministry of Education, Key Laboratory of Oasis Town and Mountain-basin System Ecology of Xinjiang Production and Construction Corps, College of Life Sciences, Shihezi University, Shihezi 832003, China; Key Laboratory of Xinjiang Phytomedicine Resource and Utilization of Ministry of Education, Key Laboratory of Oasis Town and Mountain-basin System Ecology of Xinjiang Production and Construction Corps, College of Life Sciences, Shihezi University, Shihezi 832003, China; State Key Laboratory of Plant Cell and Chromosome Engineering, Institute of Genetics and Developmental Biology, Chinese Academy of Sciences, Beijing, China; Key Laboratory of Xinjiang Phytomedicine Resource and Utilization of Ministry of Education, Key Laboratory of Oasis Town and Mountain-basin System Ecology of Xinjiang Production and Construction Corps, College of Life Sciences, Shihezi University, Shihezi 832003, China; Key Laboratory of Xinjiang Phytomedicine Resource and Utilization of Ministry of Education, Key Laboratory of Oasis Town and Mountain-basin System Ecology of Xinjiang Production and Construction Corps, College of Life Sciences, Shihezi University, Shihezi 832003, China; Key Laboratory of Xinjiang Phytomedicine Resource and Utilization of Ministry of Education, Key Laboratory of Oasis Town and Mountain-basin System Ecology of Xinjiang Production and Construction Corps, College of Life Sciences, Shihezi University, Shihezi 832003, China; Key Laboratory of Xinjiang Phytomedicine Resource and Utilization of Ministry of Education, Key Laboratory of Oasis Town and Mountain-basin System Ecology of Xinjiang Production and Construction Corps, College of Life Sciences, Shihezi University, Shihezi 832003, China

## Abstract

Alfalfa is one of the most economically valuable forage crops in the world. However, molecular cytogenetic studies in alfalfa lag far behind other cash crops and have reached a bottleneck. Here, we developed a novel chromosome identification system by designing 21 oligo probes in specific regions of each chromosome, which can be used as a barcode to simultaneously distinguish all chromosomes in a cell. Using this system, we revealed the chromosome karyotype features and evolutionary differences among 10 cultivated alfalfa varieties. Interestingly, we also found two chromosomal variation types, i.e. aneuploidy and large chromosomal segment deletions in the seeds of three alfalfa varieties. Variation frequency analysis showed that only 7/173 seeds in those three alfalfa varieties had chromosome aberrations, which indicated that the inheritance and meiosis of alfalfa had evolved to a relatively stable state. Remarkably, 4/7 variation seeds were chromosome 2 aberrations, suggesting that chromosome 2 appears to be more susceptible to natural chromosomal aberrations than other chromosomes during inheritance. DNA sequence variation analysis showed that the difference of presence and absence variations (PAVs) among homologous copies of chromosome 2 was larger than that of the other seven chromosomes. We suggest that such large PAV divergence among homologous copies may provide the physical basis for natural chromosome 2 aberrations propensity. Our study provides a valuable and efficient tool for alfalfa’s molecular cytogenetics and sheds new insights into the propensity for natural chromosome aberrations during autopolyploid inheritance.

## Introduction

Karyotype has long been considered as the most general description of the basic genetic makeup in eukaryotic species, whose basic components are the number, morphology, and size of all chromosomes in the nucleus [1]. Karyotypic variation usually refers to changes in the number and structure of chromosomes, which are closely related to genetic diversity, adaptation, and genomic differentiation among related species [2, 3]. Karyotype variation analysis mainly rely on the identification of individual chromosome. However, individual chromosome identification is still a great challenge for many non-model plants, especially those with complex polyploidy genomes and/or many small chromosomes [1].

Chromosome banding and fluorescence *in situ* hybridization (FISH) were the two landmark techniques for chromosome identification and karyotyping analysis. Chromosome banding, particularly G-banding, was widely used in mammalian karyotyping, but is not applicable to most plants. Only a few plants with large chromosomes can be identified using the chromosome banding technique, and further chromosome analysis requires extensive research experience [4–6]. FISH was a universal tool for chromosome identification and karyotyping in both animals and plants [7, 8]. In particular, chromosome-specific probes are one of the most important factors limiting the application of FISH in chromosome research [8]. Repetitive sequences, bacterial artificial chromosome (BAC) clones, and genomic DNA were the most popular FISH probes in the past few decades [9–12]. However, screening for repetitive sequences and BACs that can be used to specifically label each chromosome was technically challenging and time-consuming [13, 14], while genomic DNA probes can only track chromosomes at the genomic scale and cannot be used for individual chromosome identification [15, 16]. Each of these three probe types has its own disadvantages, for instance, repetitive sequences evolve rapidly and vary greatly among closely related species, which cannot be used as reliable chromosome-specific markers for comparative karyotype analysis; BACs often contain repetitive sequences, which easily generate background signals that affect cytological analyses in FISH experiments; genomic DNA probes are difficult to be used to distinguish chromosomes among species with close evolutionary relationships, and are unable to trace and identify individual chromosomes [8, 17, 18].

The recent emergence of new-generation probe types based on oligonucleotides (oligos) has dramatically improved the efficiency and accuracy of FISH technology for chromosome identification and karyotype analysis [18–20]. Oligo-based probes can track arbitrary chromosomes or chromosomal segments by artificially designed in conserved DNA regions via bioinformatics approaches, and are universally applicable among plant species that diverged >15 million years ago [1, 21]. This probe type can be developed for any plant species with a sequenced genome. Currently, oligo-FISH probes have been successfully applied to investigate karyotypes, chromosome aberrations, and evolution in a wide range of plant species [2, 22–28].

The cultivated alfalfa (*Medicago sativa* L., 2n = 4x = 32), as a perennial herbaceous legume, is widely used in the fields of livestock feed, edible vegetables, and medicinal herb, which is one of the most important economic crops in the world [29, 30]. Due to its adaptability, high and stable yield, and good nutritional value, it has long been as the ‘Queen of Forage’ in livestock production [31]. However, cultivated alfalfa is generally a complex autotetraploid with small and uniform chromosome shape and size (~2–3 μm), which hindered the detailed chromosome analyses in cultivated alfalfa, resulting in its cytogenetic research lagging far behind other crop species [32]. However, advances in molecular cytogenetic techniques based on FISH, has provided new opportunities to overcoming these limitations of cytogenetic research in plant species with this type of chromosome [8]. In the past three decades, various types of probes, such as repetitive sequences, BAC, and genomic DNA, have been widely used in chromosome identification, karyotype analysis, and evolution in alfalfa, which has greatly promoted the development of molecular cytogenetics in alfalfa [33–37]. However, the shortcomings and limitations of the existing probe types have restricted the further development of chromosome research in alfalfa, and the development of molecular cytogenetics in alfalfa has reached a bottleneck.

We applied the emerging oligo-based FISH technique to chromosome research in alfalfa for the first time. A set of 100 800 oligos from the single-copy sequences located at 21 specific chromosomal regions in the alfalfa genome were designed and synthesized as FISH probes. Those oligo-based probes produced 21 distinct FISH signals, which can be used as a ‘barcode’ to simultaneously distinguish all eight chromosomes of alfalfa in one cell. We revealed the chromosomal karyotypic features and evolutionary differences among 10 cultivated alfalfa varieties using oligo-based barcode probes, and demonstrated that the oligo-based barcode probes can be used as a universal and efficient chromosome identification system and are not affected by DNA polymorphism existing among different alfalfa varieties. Using this system, we unexpectedly found large chromosomal segment deletions and aneuploid variation types concentrating on chromosomes 2, 3, and 6 in seeds of three alfalfa varieties, and further investigated chromosomal variation frequency and chromosome aberrations propensity in the seeds of autotetraploid cultivated alfalfa. We demonstrate that this efficient Oligo-FISH barcode chromosome identification system is a powerful tool for chromosomal identification, aberrations, and karyotyping studies in alfalfa, and will be crucial for future investigation of chromosome evolution and diversification in the genus *Medicago* L.

## Results

### Development of oligo-FISH barcode probes for chromosome identification in *M. sativa* L.

**Figure 1:**
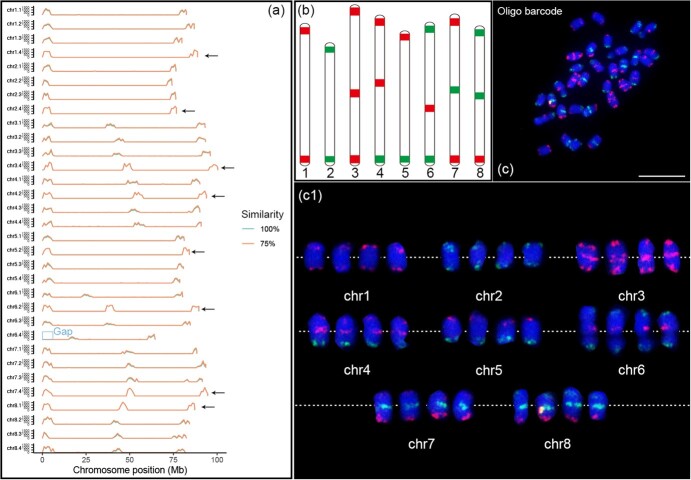
Development of oligo-FISH barcode probes for alfalfa’s chromosome identification. (a) The sequences of 21 oligo probes were mapped to the genome of autotetraploid cultivated alfalfa XinJiangDaYe. The x- and y-axes show the chromosome positions (Mb) and number of oligo sequences mapped to homologous copies, respectively. All chromosomes were divided into 1-Mb windows, and the number of oligos was counted for each window. Arrows indicate the longest homologous copies of chromosomes 1–8 used for oligo probes design. (b) Predicted positions of oligo-FISH signals on eight alfalfa chromosomes. Oligos were selected from a total of 21 chromosomal regions. The eight chromosomes can be distinguished from each other based on number and location of the FISH signals. (c) FISH mapping of the 21 oligo probes in autotetraploid cultivated alfalfa XinJiangDaYe (2n = 4x = 32). (c1) Homologous chromosomes were digitally excised from the same image of (c) and magnified 2-fold. The white dotted line represents the position of centromeres on the chromosome. Scale bars, 10 μm.

To simultaneous identification of all eight alfalfa chromosomes in the same metaphase cells. We selected 21 different regions on the eight chromosomes based on the autotetraploid alfalfa XinJiangDaYe reference genome [30]. Chromosome copy that shows the longest assembled size was used for the oligo design (Fig. 1a, shown by black arrow). The oligo sequence derived from these 21 different regions was synthesized and subsequently prepared as probes with either red or green fluorescence modifications (Table 1 and S1). These 21 different regions oligo probes were designed to produce 21 distinct FISH signals, which can be used as a barcode to uniquely label each of the eight alfalfa chromosomes (Fig. 1b). To ensure that each probe produces a uniform FISH signal intensity, each probe was composed of 4800 sequences at a density of ~1 oligo/kb. Finally, the barcode probe consisted of 100 800 oligos. Among the eight chromosomes, shorter chromosomes (1, 2 and 5) were designed with two probe markers located at both ends of the chromosome, while longer chromosomes (3, 4, 6, 7 and 8) were designed with three probes located in the middle and both ends of the chromosome (Fig. 1b, Table 1). To ensure that the signals generated by each probe can be distinguished, the distance between each probe was at least 35.8 Mb (Table 1).

**Table 1:** Characterizations of oligo probes designed based on the *M. sativa* genome.

*M. sativa* chromosome	FISH probe	Start position (bp)	Stop position (bp)	Region length (bp)	Number of oligos	Density (oligos/kb)
1	Ms1.1	0	4 440 780	4 440 780	4800	1.08
1	Ms1.2	84 374 820	88 815 615	4 440 795	4800	1.08
2	Ms2.1	0	3 837 500	3 837 500	4800	1.25
2	Ms2.2	72 912 500	76 750 018	3 837 518	4800	1.25
3	Ms3.1	0	5 020 726	5 020 726	4800	0.96
3	Ms3.2	45 186 534	50 207 260	5 020 726	4800	0.96
3	Ms3.3	95 393 794	100 414 524	5 020 730	4800	0.96
4	Ms4.1	0	4 697 371	4 697 371	4800	1.02
4	Ms4.2	51 671 081	56 368 452	4 697 371	4800	1.02
4	Ms4.3	89 250 049	93 947 428	4 697 379	4800	1.02
5	Ms5.1	0	4 208 274	4 208 274	4800	1.14
5	Ms5.2	79 957 206	84 165 483	4 208 277	4800	1.14
6	Ms6.1	0	4 478 960	4 478 960	4800	1.07
6	Ms6.2	35 831 680	40 310 640	4 478 960	4800	1.07
6	Ms6.3	85 100 240	89 579 199	4 478 959	4800	1.07
7	Ms7.1	0	4 732 886	4 732 886	4800	1.01
7	Ms7.2	47 328 860	52 061 746	4 732 886	4800	1.01
7	Ms7.3	89 924 834	94 657 719	4 732 885	4800	1.01
8	Ms8.1	0	4 362 117	4 362 117	4800	1.10
8	Ms8.2	43 621 170	47 983 287	4 362 117	4800	1.10
8	Ms8.3	82 880 223	87 242 343	4 362 120	4800	1.10

We further compared the homologies of 21 oligo barcode probe sequences among four sets of homologous chromosome copies in autotetraploid alfalfa XinJiangDaYe. The results showed that the probes maintained a consistent distribution pattern and a high level of similarity on the four homologous copies of all chromosomes except chromosome copy 6.4 (Fig. 1a, Table S2). Only 2 of 4800 oligo sequences of the Ms6.1 probe (located at 0–4.5 Mb of chr6.2) were aligned to chromosome homologous copy 6.4 (Fig. 1a, Table S2). We speculate that there may be chromosomal deletions or genome assembly gaps in the corresponding region of chr6.4. We then labeled the oligo probe according to the predicted patterns (Fig. 1b) using fluorescent dyes FAM-green or TAMRA-red, and hybridized to the metaphase chromosome prepared from Xinjiang Daye. The green and red FISH signals derived from those 21 oligo probes formed a barcode that uniquely labeled the eight chromosomes (Fig. 1c and c1), which matched the previously designed barcode pattern (Fig. 1b). The Ms6.1 probe also produced FISH signals on all four homologous copies of chromosome 6 (Fig. 1c1), suggesting the presence of a genome assembly gap in the corresponding region of the chromosome homologous copy 6.4.

### Chromosome identification and comparative karyotyping in different autotetraploid cultivated alfalfa varieties

**Figure 2:**
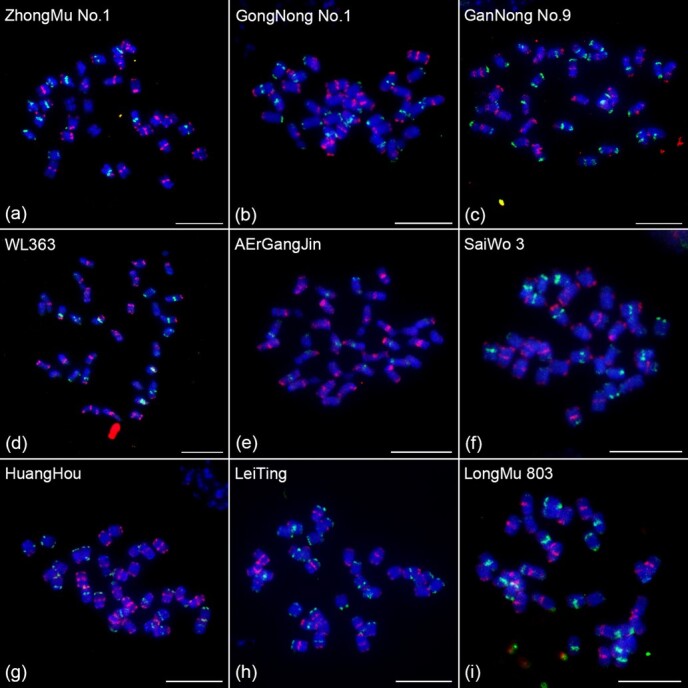
Chromosome identification in different alfalfa varieties using oligo-FISH barcode probes. FISH assays of the oligo-FISH barcode probes on metaphase chromosomes prepared from (a) ZhongMu No. 1, (b) GongNong No. 1, (c) GanNong No. 9, (d) WL363, (e) AErGangJin, (f) SaiWo 3, (g) HuangHou, (h) LeiTing, (i) LongMu 803. Scale bars, 10 μm.

To examine the universal applicability of this barcode chromosome identification system in autotetraploid alfalfa varieties. Nine different autotetraploid cultivated alfalfa varieties were selected for FISH assays. The oligo barcode probes were hybridized to the metaphase chromosome prepared from nine autotetraploid cultivated alfalfa varieties, respectively. We observed a nearly identical FISH signal pattern on chromosomes from nine different autotetraploid cultivated alfalfa varieties as those in XinJiangDaYe (Fig. 2), suggesting that this barcode chromosome identification system was universally applicable in autotetraploid alfalfa and was not affected by DNA polymorphisms existing among different alfalfa varieties.

**Figure 3:**
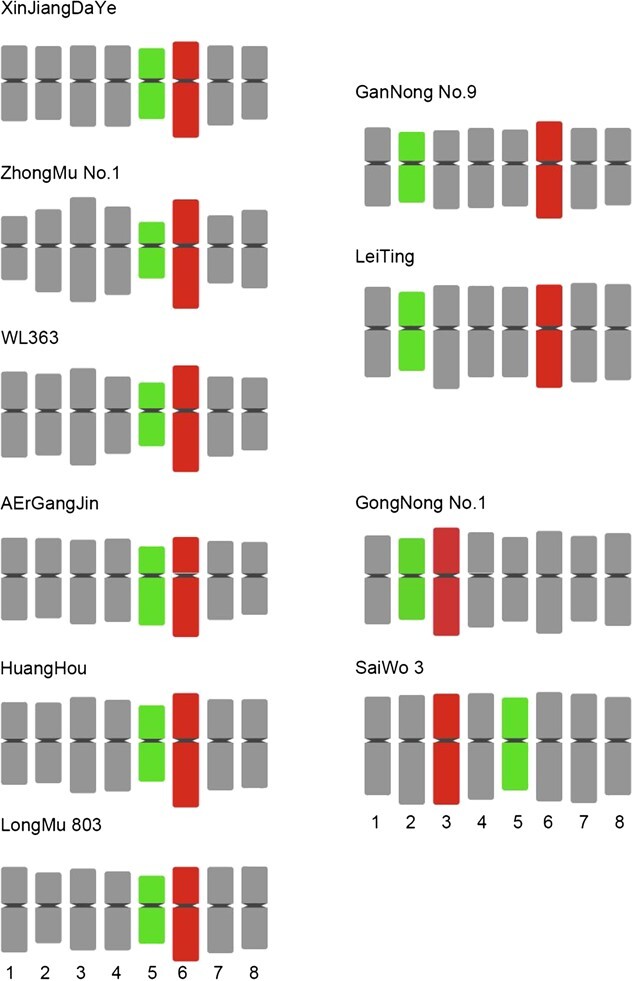
Schematic illustration of chromosome karyotype in different alfalfa varieties. The relative length and arm ratio of each chromosome in different alfalfa varieties were drawn based on the FISH results and karyotype data. The longest and shortest chromosomes are represented by rectangles with different colors.

Unambiguous identification of all chromosome in the same metaphase cell enabled us to develop karyotypes based on individually identified chromosomes. Comparative karyotype analysis showed that all the chromosomes in 10 alfalfa cultivars are metacentric (1.01 < arm ratio < 1.70), and there are no particularly long or short chromosomes (the longest chromosome was only ~1.5 times longer than the shortest chromosome) (Table S3). Among 10 cultivated alfalfa varieties, the chromosome karyotypes of XinJiangDaYe, Zhongmu No. 1, WL363, AErGangjin, Huanghou, and Longmu803 were relatively similar, in which the longest was chromosome 6 and the shortest was chromosome 5; the chromosome karyotypes of Gannong No. 9 and Leiting were more similar (the longest was chromosome 6 and the shortest was chromosome 2); and the chromosome karyotypes of Gongnong No. 1 and Saiwo3 were more similar, in which the longest was chromosome 6, but the shortest chromosome being different (i.e. chromosome 2 and 5) (Fig. 3).

### Characterization of aneuploids in cultivated alfalfa varieties using oligo-FISH barcode chromosome identification system

**Figure 4:**
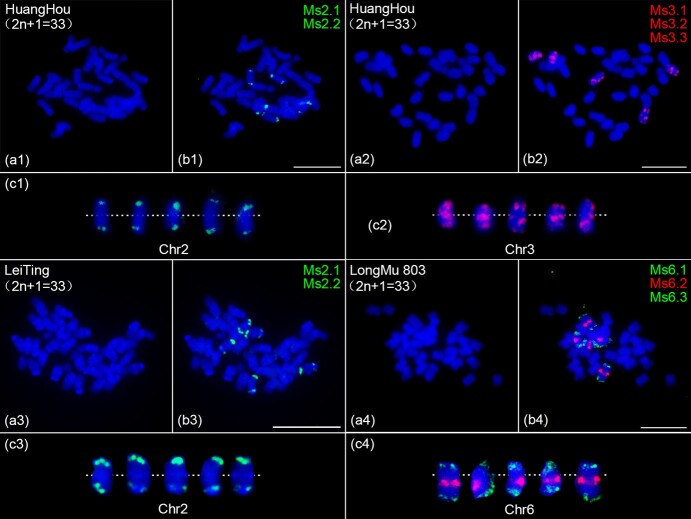
Identification of aneuploidy in alfalfa varieties. (a1–a4) DAPI-stained chromosomes from a metaphase cell in HuangHou, LeiTing, and LongMu 803, respectively. (b1, b3) FISH assays of the chr2 barcode probes on metaphase chromosomes prepared from HuangHou and LeiTing. (c1, c3) Chromosome 2 homologous copies were digitally excised from the same image of (b1, b3) and magnified 2-fold. (b2, b4) FISH assays of the chr3 and chr6 barcode probes on metaphase chromosomes prepared from HuangHou and LongMu803, respectively. (c2, c4) Chromosome 3 and 6 homologous copies were digitally excised from the same image of (b1, b3) and magnified 2-fold. The white dotted line represents the position of centromeres on the chromosome. Scale bars, 10 μm.

During the cytological analysis of 10 alfalfa cultivars (at least 10 seeds were analyzed for each cultivated alfalfa varieties), we unexpectedly found aneuploids with 33 chromosomes rather than 32 chromosomes in the seeds of Huanghou, Leiting, and Longmu 803 cultivated alfalfa varieties (Fig. S1a–d). To further characterize the chromosome composition of these three aneuploid cultivated alfalfa varieties, we then performed FISH analyses in three aneuploid cultivated alfalfa varieties seeds using chromosome-specific barcode probes, respectively. We found two types of aneuploidy in cultivated alfalfa varieties Huanghou, one with five homologous copies of chromosome 2 (Fig. 4a1–c1), and the other with five homologous copies of chromosome 3 (Fig. 4a2–c2). In cultivated alfalfa varieties Leiting, we found an aneuploidy type similar to Huanghou, which also has five homologous copies of chromosome 2 (Fig. 4a3–c3). In cultivated alfalfa varieties Longmu 803, we found a new aneuploidy type with five homologous copies of chromosome 6 (Fig. 4a4–c4).

We were intrigued by the variation frequency of naturally occurring aneuploids during the inheritance of autotetraploid cultivated alfalfa varieties. At least 50 seeds were randomly selected from each of these three cultivated alfalfa varieties for cytological analysis to determine the variation frequency of natural aneuploid. The results showed that the frequencies of natural aneuploidy variation in seeds of Huanghou, Leiting, and Longmu 803 cultivated alfalfa varieties were only 3.33% (2/60), 3.39% (2/59), and 3.7% (2/54), respectively (Table S4). The low frequency of chromosomal variation in seeds suggests that the inheritance and meiotic of autotetraploid cultivated alfalfa varieties have evolved to be relatively stable.

### Confirmation of large chromosomal segment deletions by oligo-based chromosome painting

**Figure 5:**
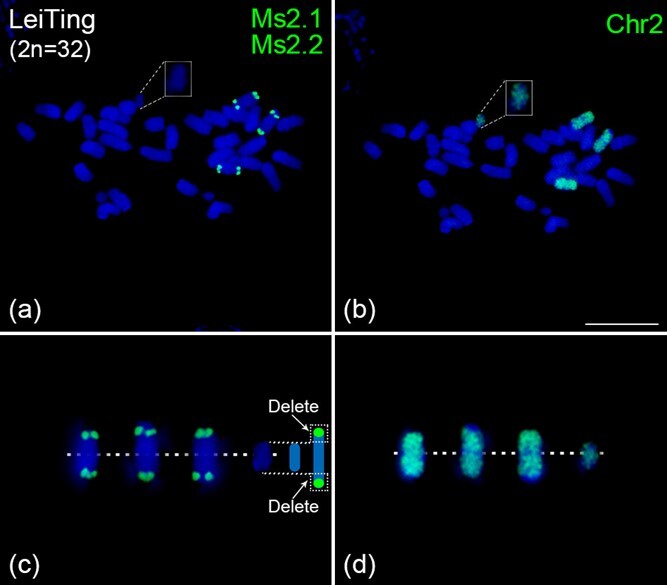
Validation of large chromosomal segment deletions by chromosome painting. (a, b) FISH assays of the chr2 barcode probes and chr2 painting probes on metaphase chromosomes prepared from LeiTing. The white rectangle shows the special homologous copy of chromosome 2 with large chromosomal segment deletions. (c) A schematic diagram explaining the occurrence of large chromosomal segment deletions at both ends of a homologous copy of chromosome 2. Chromosome 2 homologous copies were digitally excised from the same image of (a). (d) chromosome 2 homologous copies were digitally excised from the same image of (b). The white dotted line represents the position of centromeres on the chromosome. Scale bars, 10 μm.

In cultivated alfalfa varieties Leiting, we not only found an aneuploid type seed with five homologous copies of chromosome 2 (Fig. 4 a3–c3), but also unexpectedly found a seed with a total of 32 chromosomes (Fig. S1e), one of which was notably smaller than the rest (Fig. 5a). To further investigate the chromosome composition of this specific cultivated alfalfa varieties Leiting seed, we performed FISH analysis on this specific seed using chromosome-specific oligo barcode probes. The FISH results revealed an abnormality only in chromosome 2, where the chromosome 2-specific barcode probe, located at both ends of the chromosome, generated signals on just three homologous copies (Fig. 5a and c). This led us to hypothesize that this anomaly could be attributed to the formation of a new chromosome resulting from the breakage of one homologous copy of chromosome 2 at both ends (Fig. 5c), consequently, the barcode probe of chromosome 2 failed to produce signals on this newly formed chromosome homologous copy.

To test our hypothesis, we developed a whole-chromosome painting probe that can specifically identify the entire chromosome 2. A total of 95 022 oligos unique to alfalfa chromosome 2 were computationally identified and synthesized in parallel. We then performed FISH analysis on this specific seed using whole-chromosome 2 painting probe. We found that the whole-chromosome 2 painting probe not only produced signals on the three homologous copies of chromosome 2, but also produced signals on that special small chromosome (Fig. 5b and d), which is completely consistent with our hypothesis (Fig. 5c). We also further investigated the variation frequency of this large chromosomal segment deletions type in cultivated alfalfa varieties Leiting. The results show that the frequency of this chromosomal variant type is only 1.69% (1/59) (Table S4).

### DNA sequence variation of homologous chromosomes in autotetraploid alfalfa

**Figure 6:**
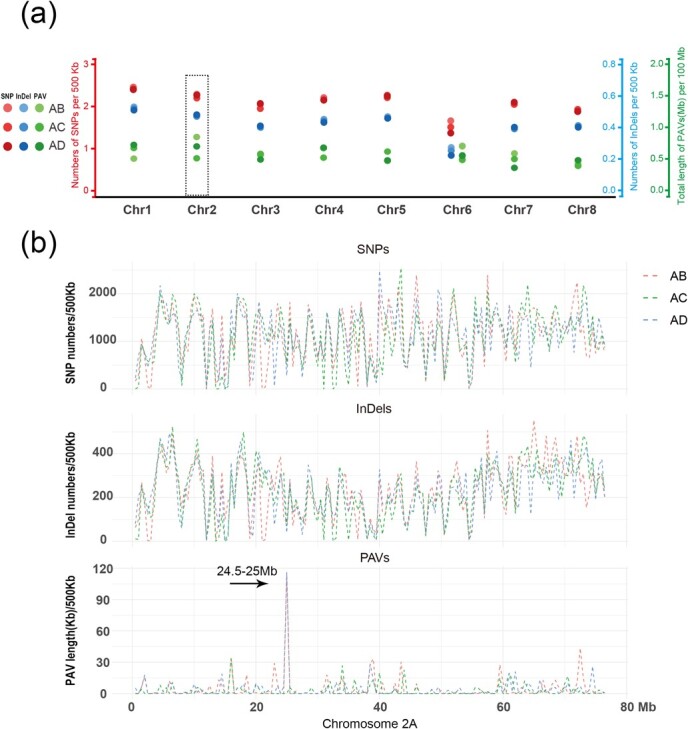
DNA sequence variation among four homologous copies of chromosome 1–8 in autotetraploid alfalfa. Distribution of SNP, InDel, and PAVS in alfalfa chromosomes. The x-axis and y-axis indicate the alfalfa chromosome and the distribution of SNP, InDel, and PAVS, respectively. SNP, InDel and PAVS are represented by different colors. The gradient colored circles represent sequence variations between the A copy and the other three homologous (B, C, and D), respectively. A, B, C, and D represent chrx.1, chrx.2, chrx.3, and chrx.4 in autotetraploid alfalfa genome. (b) Sequence variations of SNP, InDel, and PAVS on chromosome 2 of alfalfa. The x-axis and y-axis represents chromosome positions (Mb) and distributions of SNP, InDel, and PAVS on chromosome 2, respectively. Sequence differences between AB, AC, and AD homologous chromosomes are indicated by different colors, respectively. Arrows show a segment of ~120 kb of large PAV difference between homologous copies of A and B/D at 24.5–25 Mb on chromosome 2.

In this study, we performed cytological analysis on the seeds of 10 alfalfa varieties, and found that natural chromosomal aberrations (aneuploid or large chromosomal segment deletions) existed in three alfalfa varieties (Huanghou, Leiting, and Longmu803), of which chromosome 2 aberrations were detected in two alfalfa varieties (Huanghou and Leiting) (Table S4). A total of 173 seeds of the three alfalfa varieties were analyzed, and chromosomal aberrations were detected in seven seeds, of which chromosome 2 aberrations were detected in four seeds (Table S4). We are intrigued by why chromosome 2 is more susceptible to produced chromosomal aberrations than other chromosome during inheritance. Previous studies have shown that DNA sequence variation may affect the pattern of meiosis and inheritance [38,39]. We speculate whether there might be cryptic sequence variation among the four homologous copies of chromosome 2 that would make it more susceptible to produced chromosomal aberrations than the other seven chromosomes. We then examined the DNA sequence similarity (single-nucleotide polymorphisms (SNPs), InDels, and PAVs) among the four homologous copies of chromosome 1–8 using the genomic sequencing data of autotetraploid alfalfa. The results showed that no distinct differences in the density of SNPs/Indels and the total amount (Mb) of PAVs among four homologous copies of each chromosome (Fig. 6a, Table S5). Among them, the sequence variation level of SNPs/InDels density associated with chromosome 2 was in the upper middle position of the range of the variation among the 10 chromosomes (Fig. 6a, Table S5). However, it is remarkable that chromosome 2 is the shortest chromosome in the genome (~76.8 Mb), but the total amount of PAVs (~655 656 bp) between chr2A and 2B homologous copies is largest (Fig. 6a, Table S5).

We further analyzed the distribution characteristics of SNPs, InDels, and PAVs (per 500 kb) among four homologous copies of chromosome 2 and the other seven chromosomes. The results showed that the distribution of SNPs and InDels on chromosome 2 had no apparent differences from that of the other seven chromosomes, but the distribution of PAVs showed a distinct difference between chr2A, 2C and chr2B, 2D (Fig. 6b and S2). Notably, chr2A, 2C and chr2B, 2D have a PAV variation of ~120 Kb in the 24.5–25 Mb region, which is larger than the PAV variation among homologous copies of all other seven chromosomes (Fig. 6b and S2). Thus, we speculate that chromosome 2 was prone to experiencing natural chromosomal abnormalities, which were most likely caused by genetic and meiotic irregularities associated with a large number of PAV variations among homologous copies.

## Discussion

Cultivated alfalfa (*M. sativa* L.), a perennial leguminous forage, is one of the most economically valuable crops in the world, and has been widely cultivated over 80 countries with coverage exceeding 30 million hectares [30]. Although alfalfa is so important, the molecular cytogenetic research of alfalfa is far behind other cash crops, mainly due to the lack of strong chromosome identification markers. In this study, we developed a set of powerful barcode chromosome identification tools for alfalfa through genome-directed design, breaking the bottleneck of the shortage of chromosome markers in alfalfa. This set of barcode probescan simultaneously distinguish all chromosomes of autotetraploid alfalfa in one cell, and match pseudomolecules in the genome to cytological chromosomes (Fig. 1). By barcode oligo-FISH, we unexpectedly detected a haplotype assembly gap on chromosome 6.4 in autotetraploid alfalfa genome, which demonstrates the potential application value of the oligo-FISH technique in assisting genome assembly and genome quality assessment of alfalfa. Comparative FISH analysis showed that the barcode chromosome identification system has universal applicability in different autotetraploid alfalfa varieties and was not affected by DNA polymorphism among varieties (Fig. 2), which will provide a powerful tool for comparative cytological research of alfalfa in the future. Using this set of barcode probes, we dissected the chromosomal karyotype characteristics of autotetraploid alfalfa, and elucidated the degree of karyotype variation among 10 alfalfa varieties (Fig. 3, Table S3). As we know that hybridization between parents with large differences in genetic basis is more likely to produce hybrid advantage, our results can provide a molecular cytogenetic reference for the efficient utilization of hybrid advantage in alfalfa hybrid breeding. In summary, we believe that our oligo barcode chromosome identification system will be a valuable and efficient tool to significantly contribute to the facilitation of alfalfa’s molecular cytogenetic investigations, and further application will open new avenues for comprehending the chromosome origin and evolution and promoting chromosome engineering breeding in the genus *Medicago*.

Polyploidy usually refers to an individual with three or more complete sets of chromosomes in a somatic cell. Polyploids are very common in plants, and it has been estimated that ~47%–70% of flowering plants are descended from polyploid ancestors [40, 41]. Polyploids have the advantage of more vigorous and better adaptability, and the disadvantage of producing chromosomally variable gametes and progeny [42–44]. For cultivated alfalfa, typically recognized as a complex autotetraploid, it is uncertain whether offspring of autotetraploid cultivated alfalfa exhibit chromosomal variations, and if so, what the categories are of such variations. Our study provides cytological evidence for the type and frequency of chromosomal variation in autotetraploid cultivated alfalfa. We found two types of variation in autotetraploid cultivated alfalfa seeds, i.e. aneuploidy and large chromosomal segment deletions (Fig. 4 and 5). However, the frequency of chromosomal variation was very low (only 7/173 seeds had chromosomal variation), which indicated that the heredity and meiosis of autotetraploid alfalfa had evolved to a relatively stable state. This finding reveals the genetic evolution status of autotetraploid alfalfa and the phenomenon of chromosome variation in offspring after irregular inheritance, which is helpful for us to better understand the inheritance and variation of alfalfa.

Autopolyploid plants may produce offspring with chromosomal variations during inheritance. However, each species has its own unique chromosome composition, and there is still unclear about which chromosome(s) will undergo variation and whether there is a propensity for such variation. In this study, we found that four of the seven variable seeds in autotetraploid alfalfa were chromosome 2 variations (Table S4). Previous studies have shown that DNA sequence variation was associated with meiosis and inheritance variability [38, 45]. DNA sequence variation analysis of homologous chromosomes in autotetraploid alfalfa showed that chromosome 2, as the smallest chromosome in the genome, had the largest PAV amounts among homologous copies of chromosome 1–8 (Fig. 6 and S2, Table S5). We hypothesize that large PAV differences among homologous copies are likely to affect meiosis and genetic stability of alfalfa chromosome 2, which leads to the susceptibility of alfalfa chromosome 2 to undergo chromosomal structural and numerical variation during inheritance. Based on our results, we suggest that such large PAV divergence among homologous copies may provide the physical basis for natural chromosome aberrations propensity in autopolyploid species. Moreover, we also found two seeds with chromosome 6 number variation and one seed with chromosome 3 number variation except for chromosome 2 (Table S4). DNA sequence analysis showed that the number of SNPs and Indels among the four homologous copies of chromosome 6 was the lowest (Fig. 6, Table S5). We speculated that the high similarity among the four homologous copies of chromosome 6 might be related to chromosome 6 number variation during inheritance. For chromosome 3, we did not find significant differences in SNPs, Indels, and PAVs among its four homologous copies compared to the other chromosomes (Fig. 6, Table S5). We tend to believe that the seeds with chromosome 3 number variations are randomly generated with a very small probability during inheritance, and are not related to DNA sequence variation among homologous copies. As we know, chromosomal variations are likely to fail to form seeds, and even if seeds are formed, they are likely to grow and develop abnormally or even die, but we found that the seeds of the chromosome variations on chromosomes 2, 3, and 6 all grew and developed normally, which suggests that autotetraploid alfalfa has reduced the impact of chromosomal variations through some unknown mechanism. It will be interesting to further explore the mechanisms of how polyploidy reduces the effects of chromosomal variation on growth and development. Together, our study reveals chromosomal aberrations in autotetraploid cultivated alfalfa during inheritance, and provides a novel insight into the propensity of natural chromosome aberration, which will contribute to the understanding of inheritance and evolutionary paths in polyploid *Medicago* species.

## Materials and methods

### Plant material and chromosome preparation

Ten cultivated alfalfa varieties, including XinJiangDaYe, Huanghou, Leiting, Longmu803, WL363, Saiwo3, Zhongmu No. 1, Gongnong No. 1, Gannong No. 9, and Aergangjin were used in FISH mapping in this study. All materials were planted in the greenhouse of Shihezi University. The metaphase chromosomes from root tips were prepared based on the published procedure [25]. Briefly, the root tips were collected from those 11 cultivated alfalfa varieties and treated with cycloheximide for 2 h for 1.5 h to enrich the metaphase cells. Then, root tips were fixed in fixative solution (3 ethanol:1 acetic acid) and stored at −20°C. An enzymatic solution with 4% cellulase (w/v) (Yakult Pharmaceutical, Tokyo, Japan), 2% pectinase (w/v) (Plant Media, Pittsburgh, PA, USA), and 2% (w/v) cellulase ‘Onozuka’ RS (Yakult Pharmaceutical, Tokyo, Japan) was used to digest the root tips for 2 h at 37°C. Then, the metaphase chromosome was prepared followed published protocols [25]. Chromosome slides were stored at −80°C until FISH experiments.

### Development of oligo-based barcode probe

The Oligo-based barcode probe sets were designed based on the reference genome of autotetraploid alfalfa XinJiangDaYe [30] using Chorus2 software (https://github.com/zhangtaolab/Chorus2) following previous procedures [46]. Briefly, we selected the longest assembled size of the four homologous copies of chromosome 1–8 in autotetraploid alfalfa for oligo design, and the chromosome sequences were divided into 45-bp oligo sequences with the default parameter ‘-l 45 -homology 75 -step 5’ via bioinformatics approaches. Then, the repetitive sequences-related oligos were filtered using the genomic sequence data of XinJiangDaYe. For the barcode oligo probes, oligos from 21 chromosomal regions were selected to create a barcode for all eight alfalfa chromosomes. Each selected region spans 3.8–5.0 Mb, and contains ~4800 oligos. Finally, a total of 100 800 oligos were selected for chromosome 1–8. These oligos were synthesized by MYcroarray (Ann Arbor, MI, USA) and labeled with FAM-green or TAMRA-red (direct) primers (Table S1) following published polymerase chain reaction protocol [47].

### Oligo-FISH and karyotyping

The oligo-FISH procedure was conducted as described previously [14]. The hybridization mixture consisted of 10 μl of 100% deionized formamide, 2 μl of 20xSSC, 4 μl of a 50% dextran sulfate solution, and ~100 ng of oligo probe; after being denatured at 90°C for 5 min, the hybridization mixture was applied directly to denaturated chromosome slides and incubated for 1 day at 37°C. After hybridization, chromosome slides were counterstained with DAPI (Vector Laboratories, USA). Chromosome and probes signal were directly examined under an Olympus BX53 fluorescence microscope. FISH images were captured using an Olympias DP80 CCD camera with cellSens Dimension 1.9 software. The final FISH images were adjusted and merged using Image-Pro Plus and Adobe Photoshop CC software. For chromosome karyotyping assay, 10 complete mitotic metaphase chromosome spreads from 11 cultivated alfalfa varieties, showing no apparent chromosomal morphological distortion, were chosen for measurements of chromosome length (tl = L + S), arm ratio (AR = L/S), and relative length (RL = tl/TL × 100). Chromosomes were classified based on arm ratio type [48].

### Sequence variation analysis

The genomic data were used to identify SNPs， insertion–deletion mutations (InDels, 1–200 bp), and PAV (>200 bp) [30]. The sequence variation analysis among four homologous copies of chromosome 1–8 were conducted using NUCmer (https://github.com/mummer4/mummer). Identification of SNPs, InDels, and PAVs was performed based on the results generated by NUCmer using SyRI [49]. Data visualization was conducted using ggplot2 (https://ggplot2-book.org/;  https://www.r-project.org/).

## Supplementary Material

Web_Material_uhae266

## Data Availability

All data are available in the manuscript or supplementary materials.
