# Supplementary material for: Oligo-FISH barcode chromosome identification system provides novel insights into the natural chromosome aberrations propensity in the autotetraploid cultivated alfalfa
Source: Hortic Res. 2024 Sep 20;12(1):uhae266. doi: 10.1093/hr/uhae266 (PMC11718389; doi:10.1093/hr/uhae266)
Supplement: Web_Material_uhae266 [file web_material_uhae266.zip › Fig S2.pdf]

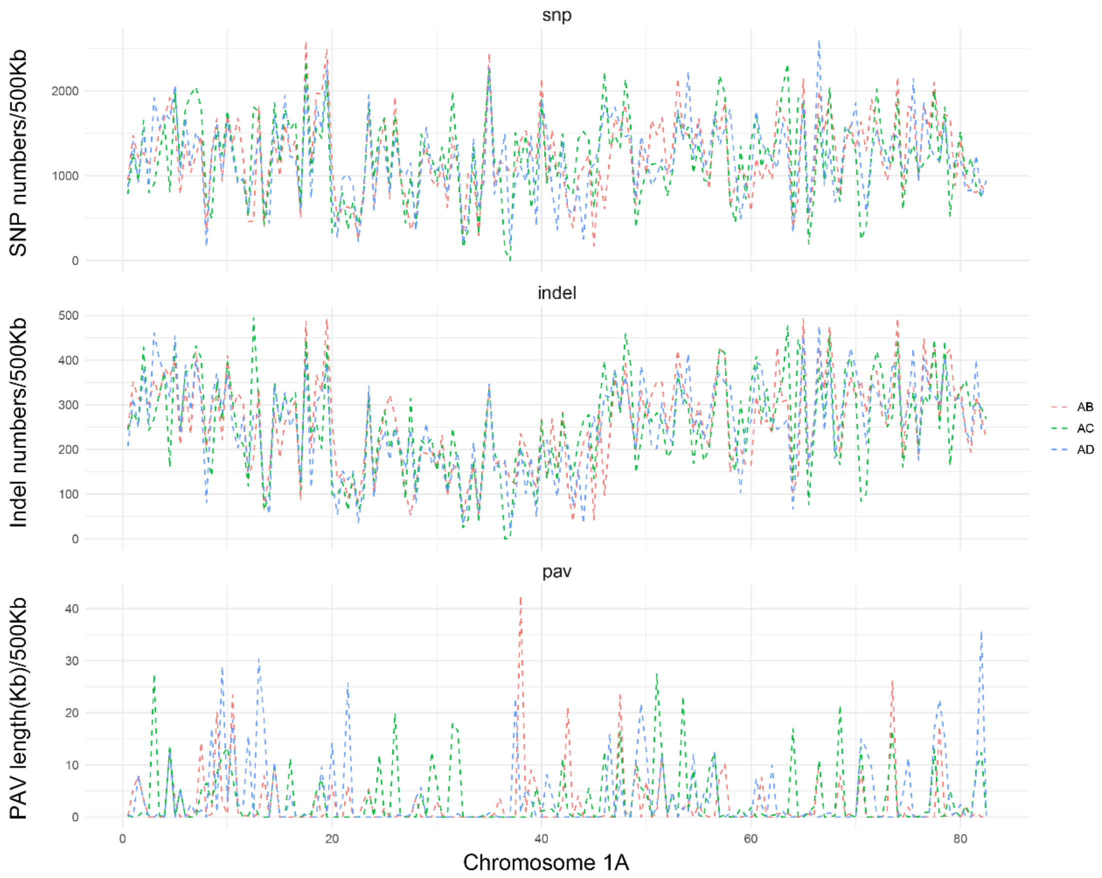
snp
SNP numbers/500Kb
2000
1000
0
indel
Indel numbers/500Kb
500
400
300
200
100
0
pav
PAV length(Kb)/500Kb
40
30
20
10
0
0
20
40
60
80
Chromosome 1A
AB
AC
AD

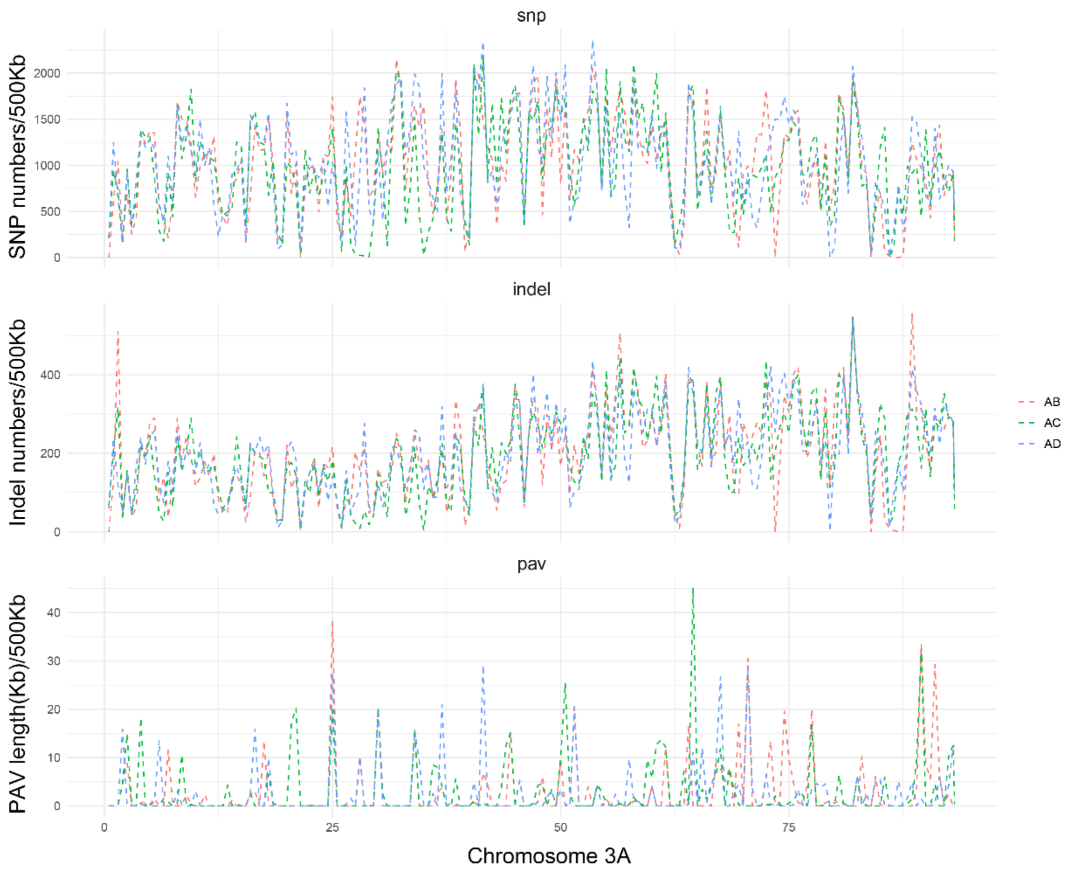

snp
SNP numbers/500Kb
0
500
1000
1500
2000
indel
Indel numbers/500Kb
0
200
400
pav
PAV length(Kb)/500Kb
0
10
20
30
40
0
25
50
75
Chromosome 3A
AB
AC
AD

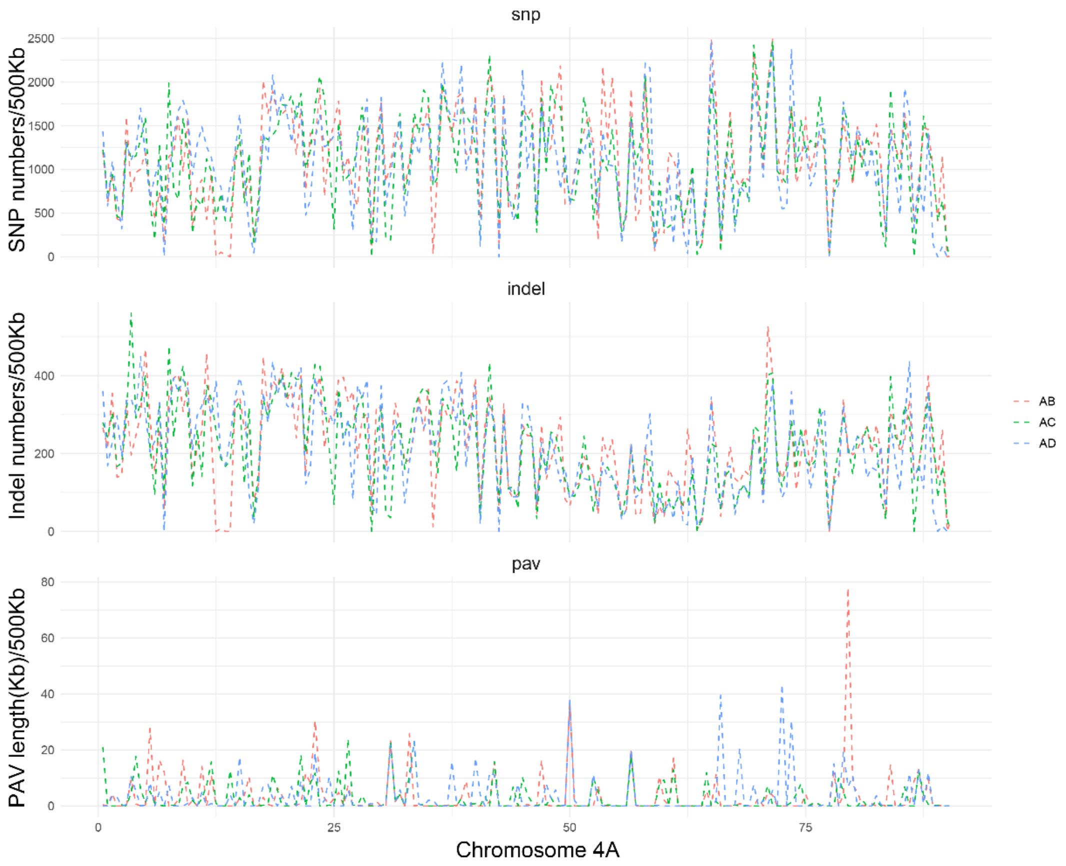

snp
SNP numbers/500Kb
2500
2000
1500
1000
500
0
indel
Indel numbers/500Kb
400
200
0
pav
PAV length(Kb)/500Kb
80
60
40
20
0
0
25
50
75
Chromosome 4A
AB
AC
AD

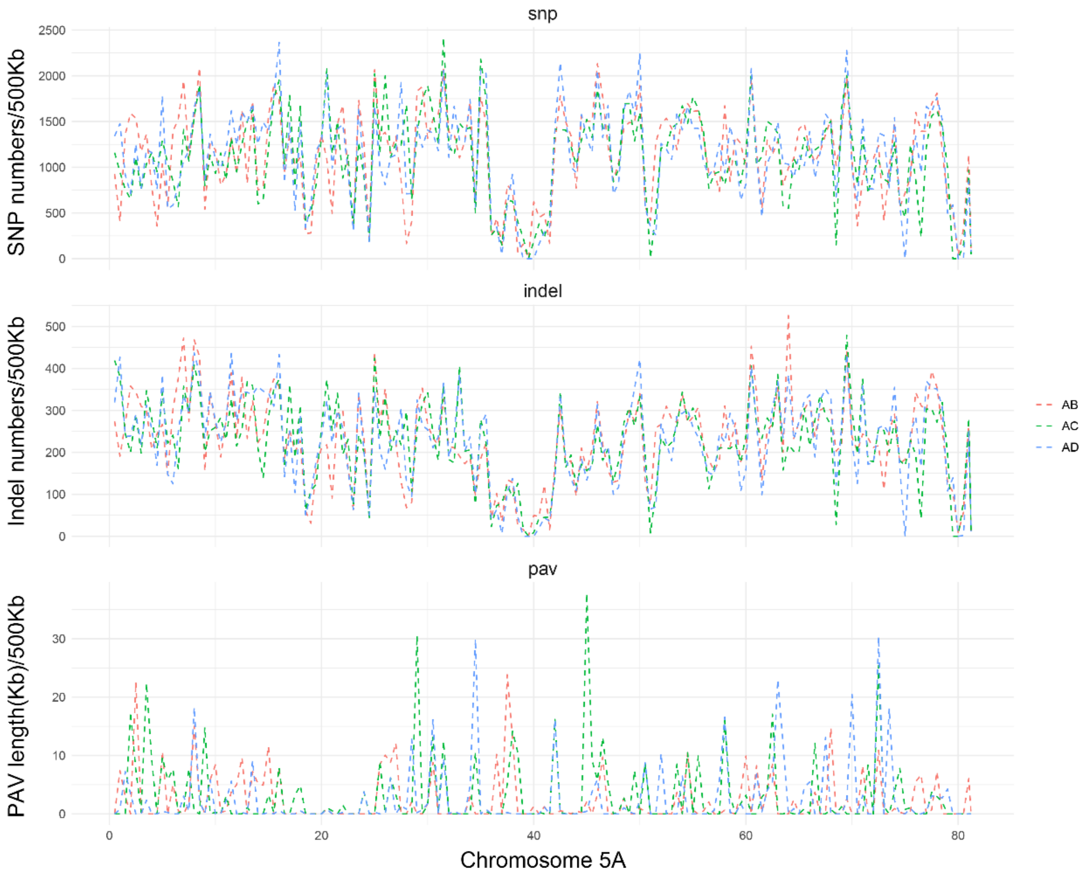
snp
SNP numbers/500Kb
2500
2000
1500
1000
500
0
indel
Indel numbers/500Kb
500
400
300
200
100
0
pav
PAV length(Kb)/500Kb
30
20
10
0
0
20
40
60
80
Chromosome 5A
AB
AC
AD

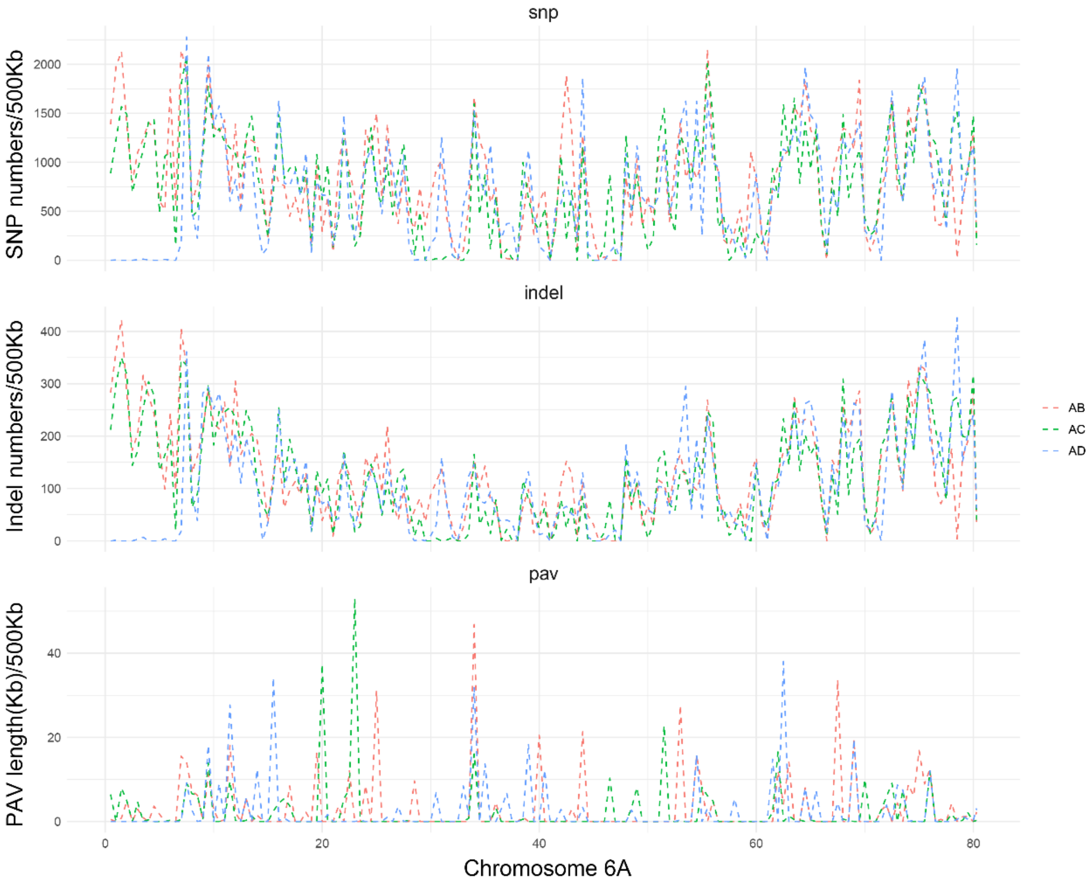
snp
SNP numbers/500Kb
2000
1500
1000
500
0
indel
Indel numbers/500Kb
400
300
200
100
0
pav
PAV length(Kb)/500Kb
40
20
0
0
20
40
60
80
Chromosome 6A
AB
AC
AD

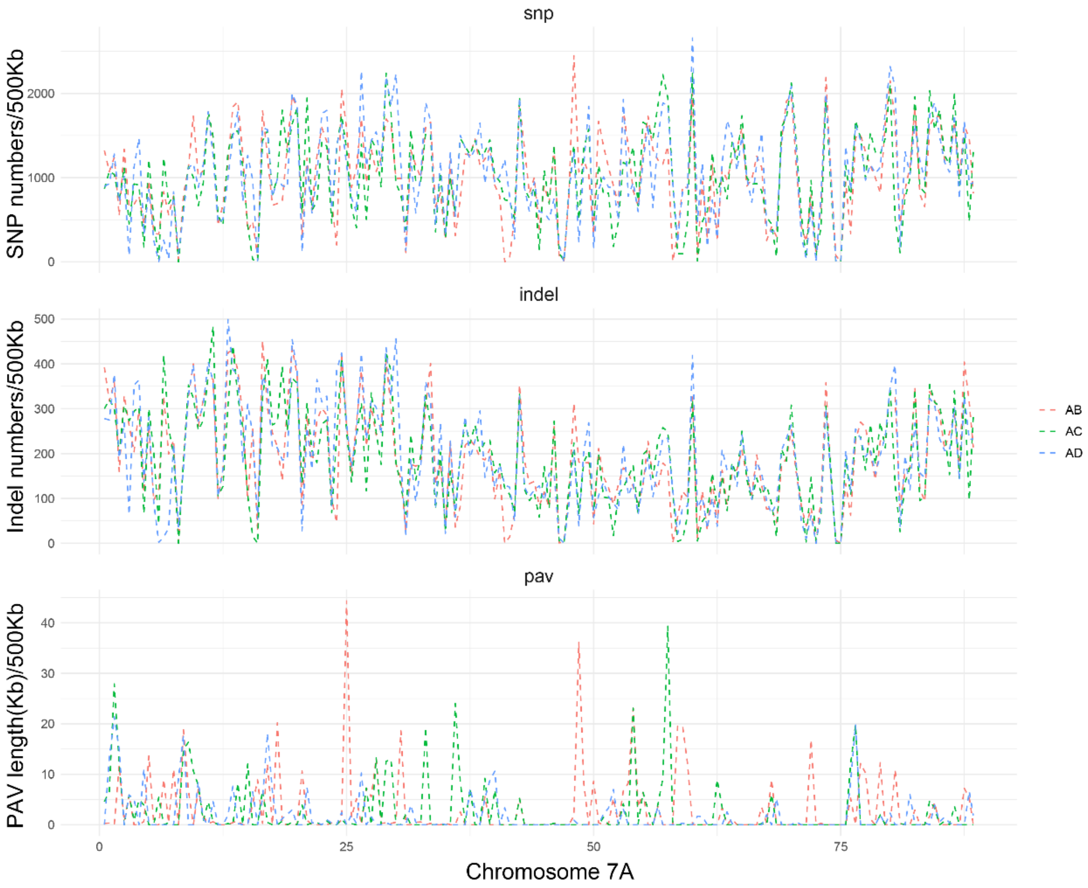
snp
SNP numbers/500Kb
0
1000
2000
indel
Indel numbers/500Kb
0
100
200
300
400
500
pav
PAV length(Kb)/500Kb
0
10
20
30
40
0
25
50
75
Chromosome 7A
AB
AC
AD

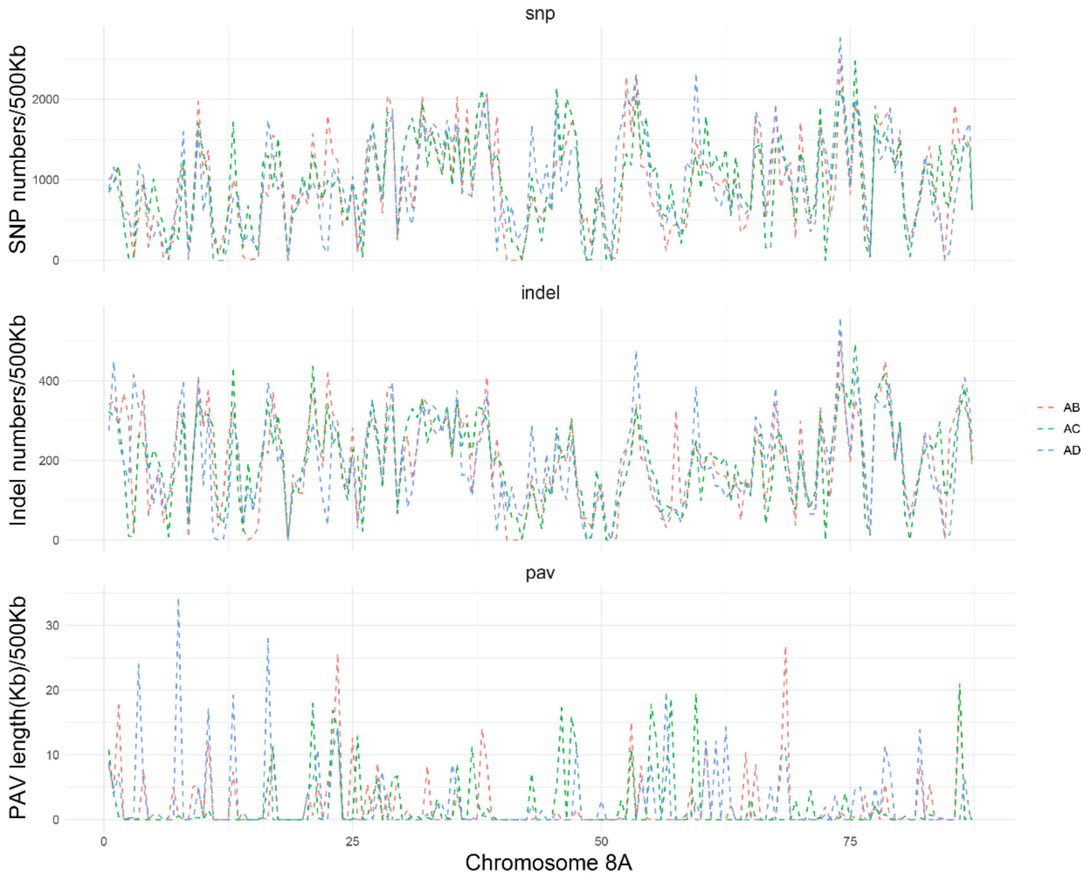

Fig S2. Analysis of sequence variation among four homologous copies of 7 chromosomes in autotetraploid alfalfa XinJiangDaYe.

The SNPs, indels, and PAVs of the largest size copy versus the other three copies in 7 chromosomes (1 and 3-8) were identified separately. The numbers of SNPs and indels and the total length of PAVs per 500 kb were calculated and plotted.
