# Supplementary material for: Oligo-FISH barcode chromosome identification system provides novel insights into the natural chromosome aberrations propensity in the autotetraploid cultivated alfalfa
Source: Hortic Res. 2024 Sep 20;12(1):uhae266. doi: 10.1093/hr/uhae266 (PMC11718389; doi:10.1093/hr/uhae266)
Supplement: Web_Material_uhae266 [file web_material_uhae266.zip › Table S1.docx]

**Table S1. Flanking primer information for each oligo probe.**

| FISH probe | Oligo flanking primers F | Sequences 5'-3' | Oligo flanking primers R | Sequences 5'-3' |
| --- | --- | --- | --- | --- |
| Ms1.1 | MF5 | CAACCACTTCATCAGTTGGA | MR5 | GGTTTGTCACCTTCAAAACA |
| Ms1.2 | MF28 | TGGTTTGAGAGGGAAAGAAC | MR28 | AAAATGTATCCAGAGCAGCC |
| Ms2.1 | MF5 | CAACCACTTCATCAGTTGGA | MR5 | GGTTTGTCACCTTCAAAACA |
| Ms2.2 | MF28 | TGGTTTGAGAGGGAAAGAAC | MR28 | AAAATGTATCCAGAGCAGCC |
| Ms3.1 | MF5 | CAACCACTTCATCAGTTGGA | MR5 | GGTTTGTCACCTTCAAAACA |
| Ms3.2 | MF17 | AAAGTGCCCGAGAGCATAGA | MR17 | TTGCGATCATTAGGAAGGAA |
| Ms3.3 | MF28 | TGGTTTGAGAGGGAAAGAAC | MR28 | AAAATGTATCCAGAGCAGCC |
| Ms4.1 | MF5 | CAACCACTTCATCAGTTGGA | MR5 | GGTTTGTCACCTTCAAAACA |
| Ms4.2 | MF19 | ACACTCAACCGTGTCATTGG | MR19 | TCCTCGCATTCCATCATGTA |
| Ms4.3 | MF28 | TGGTTTGAGAGGGAAAGAAC | MR28 | AAAATGTATCCAGAGCAGCC |
| Ms5.1 | MF5 | CAACCACTTCATCAGTTGGA | MR5 | GGTTTGTCACCTTCAAAACA |
| Ms5.2 | MF28 | TGGTTTGAGAGGGAAAGAAC | MR28 | AAAATGTATCCAGAGCAGCC |
| Ms6.1 | MF5 | CAACCACTTCATCAGTTGGA | MR5 | GGTTTGTCACCTTCAAAACA |
| Ms6.2 | MF16 | ACACCATGGACCAAAACCAT | MR16 | ATCCGTGAACCTCGTAAACG |
| Ms6.3 | MF28 | TGGTTTGAGAGGGAAAGAAC | MR28 | AAAATGTATCCAGAGCAGCC |
| Ms7.1 | MF5 | CAACCACTTCATCAGTTGGA | MR5 | GGTTTGTCACCTTCAAAACA |
| Ms7.2 | MF18 | AAATCCACGTTCTTCAACCG | MR18 | GACGATGTGTTTGGCTCCTT |
| Ms7.3 | MF28 | TGGTTTGAGAGGGAAAGAAC | MR28 | AAAATGTATCCAGAGCAGCC |
| Ms8.1 | MF5 | CAACCACTTCATCAGTTGGA | MR5 | GGTTTGTCACCTTCAAAACA |
| Ms8.2 | MF18 | AAATCCACGTTCTTCAACCG | MR18 | GACGATGTGTTTGGCTCCTT |
| Ms8.3 | MF28 | TGGTTTGAGAGGGAAAGAAC | MR28 | AAAATGTATCCAGAGCAGCC |
