# Supplementary material for: Oligo-FISH barcode chromosome identification system provides novel insights into the natural chromosome aberrations propensity in the autotetraploid cultivated alfalfa
Source: Hortic Res. 2024 Sep 20;12(1):uhae266. doi: 10.1093/hr/uhae266 (PMC11718389; doi:10.1093/hr/uhae266)
Supplement: Web_Material_uhae266 [file web_material_uhae266.zip › Table S2.docx]

**Table S2. Statistical analysis of oligo sequence similarity among homologous chromosome copies in alfalfa**

| **Ms1.1** | | | **Ms1.2** | | | **Ms2.1** | | | **Ms2.2** | | | **Ms3.1** | | | **Ms3.2** | | | | **Ms3.3** | | |
| --- | --- | --- | --- | --- | --- | --- | --- | --- | --- | --- | --- | --- | --- | --- | --- | --- | --- | --- | --- | --- | --- |
| chromosome | p=100 | p≥75 | chromosome | p=100 | p≥75 | chromosome | p=100 | p≥75 | chromosome | p=100 | p≥75 | chromosome | p=100 | p≥75 | chromosome | p=100 | p≥75 | chromosome | | p=100 | p≥75 |
| chr1.1 | 3479 | 4124 | chr1.1 | 3497 | 4049 | chr2.1 | 4174 | 4765 | chr2.1 | 3426 | 3953 | chr3.1 | 2773 | 3595 | chr3.1 | 2392 | 3338 | chr3.1 | | 3848 | 4330 |
| chr1.2 | 3404 | 4027 | chr1.2 | 3854 | 4391 | chr2.2 | 2458 | 2932 | chr2.2 | 3035 | 3548 | chr3.2 | 2695 | 3343 | chr3.2 | 1857 | 2466 | chr3.2 | | 3567 | 4075 |
| chr1.3 | 3714 | 4227 | chr1.3 | 3542 | 4015 | chr2.3 | 3219 | 3735 | chr2.3 | 3734 | 4316 | chr3.3 | 2877 | 3525 | chr3.3 | 1663 | 2340 | chr3.3 | | 3788 | 4403 |
| **Ms4.1** | | | **Ms4.2** | | | **Ms4.3** | | | **Ms5.1** | | | **Ms5.2** | | | **Ms6.1** | | | | **Ms6.2** | | |
| chromosome | p=100 | p≥75 | chromosome | p=100 | p≥75 | chromosome | p=100 | p≥75 | chromosome | p=100 | p≥75 | chromosome | p=100 | p≥75 | chromosome | p=100 | p≥75 | chromosome | | p=100 | p≥75 |
| chr4.1 | 4304 | 4852 | chr4.1 | 2118 | 3045 | chr4.1 | 3354 | 4084 | chr5.1 | 3190 | 3889 | chr5.1 | 3179 | 4072 | chr6.1 | 2215 | 2897 | chr6.1 | | 1675 | 2348 |
| chr4.3 | 3626 | 4145 | chr4.3 | 1853 | 2745 | chr4.3 | 3931 | 4669 | chr5.3 | 3328 | 4047 | chr5.3 | 2476 | 2957 | chr6.3 | 2645 | 3319 | chr6.3 | | 1162 | 1802 |
| chr4.4 | 3180 | 3796 | chr4.4 | 2409 | 3237 | chr4.4 | 2140 | 2517 | chr5.4 | 3264 | 3945 | chr5.4 | 2808 | 3486 | chr6.4 | 1 | 2 | chr6.4 | | 1106 | 1761 |
| **Ms6.3** | | | **Ms7.1** | | | **Ms7.2** | | | **Ms7.3** | | | **Ms8.1** | | | **Ms8.2** | | | | **Ms8.3** | | |
| chromosome | p=100 | p≥75 | chromosome | p=100 | p≥75 | chromosome | p=100 | p≥75 | chromosome | p=100 | p≥75 | chromosome | p=100 | p≥75 | chromosome | p=100 | p≥75 | chromosome | | p=100 | p≥75 |
| chr6.1 | 2363 | 2956 | chr7.1 | 3543 | 4002 | chr7.1 | 1731 | 2458 | chr7.1 | 2197 | 2690 | chr8.2 | 5649 | 6240 | chr8.2 | 1785 | 2454 | chr8.2 | | 2209 | 2935 |
| chr6.3 | 2174 | 2838 | chr7.2 | 4323 | 4943 | chr7.2 | 2104 | 2796 | chr7.2 | 2928 | 3829 | chr8.3 | 2295 | 2658 | chr8.3 | 2247 | 2949 | chr8.3 | | 2621 | 3360 |
| chr6.4 | 2624 | 3331 | chr7.3 | 4276 | 4743 | chr7.3 | 2794 | 3602 | chr7.3 | 2242 | 2833 | chr8.4 | 4829 | 5404 | chr8.4 | 2049 | 2769 | chr8.4 | | 1912 | 2343 |

p represents the similarity.
