# Supplementary material for: Oligo-FISH barcode chromosome identification system provides novel insights into the natural chromosome aberrations propensity in the autotetraploid cultivated alfalfa
Source: Hortic Res. 2024 Sep 20;12(1):uhae266. doi: 10.1093/hr/uhae266 (PMC11718389; doi:10.1093/hr/uhae266)
Supplement: Web_Material_uhae266 [file web_material_uhae266.zip › Table S3.docx]

**Relative length of individual chromosomes of alfafa.**

| **Chr.** | **XinJiangDaYe** | **ZhongMu No.1** | **GongNong No.1** | **GanNong No.9** | **WL363** | **AErGangJin** | **SaiWo 3** | **HuangHou** | **LeiTing** | **LongMu 803** |
| --- | --- | --- | --- | --- | --- | --- | --- | --- | --- | --- |
| 1 | 11.88±1.32 | 10.41±0.84 | 12.06±2.27 | 12.26±1.04 | 12.97±1.04 | 12.98±1.89 | 11.90±2.12 | 11.43±0.98 | 11.89±1.43 | 13.22±1.76 |
| 2 | 11.66±1.64 | 11.86±1.82 | 10.99±2.22 | 10.98±1.62 | 12.36±1.17 | 12.52±2.01 | 11.93±2.25 | 11.13±1.13 | 10.26±0.91 | 10.86±0.76 |
| 3 | 12.83±1.34 | 14.50±1.86 | 14.74±2.43 | 12.29±1.48 | 14.69±2.31 | 13.19±3.60 | 13.41±2.52 | 13.33±2.30 | 13.63±1.74 | 12.61±1.45 |
| 4 | 12.88±1.78 | 12.94±1.02 | 12.87±2.22 | 12.44±1.84 | 11.52±1.33 | 13.10±1.21 | 12.90±2.02 | 12.72±1.54 | 13.02±2.27 | 12.12±1.68 |
| 5 | 11.09±1.60 | 9.73±1.22 | 11.40±1.79 | 11.96±1.05 | 9.35±0.89 | 9.95±0.60 | 11.16±2.44 | 10.45±1.14 | 11.87±1.73 | 10.33±1.02 |
| 6 | 15.35±2.12 | 15.82±3.05 | 13.93±3.65 | 15.29±1.42 | 16.19±1.78 | 14.61±1.19 | 13.24±2.96 | 15.98±2.05 | 13.61±2.07 | 14.69±1.95 |
| 7 | 12.69±1.64 | 12.97±2.09 | 11.49±2.81 | 12.76±1.61 | 12.06±1.21 | 12.33±2.57 | 13.31±1.86 | 12.58±1.20 | 13.08±2.63 | 13.31±2.03 |
| 8 | 11.62±1.62 | 11.76±1.21 | 12.53±1.87 | 12.02±1.46 | 10.87±1.92 | 11.33±0.80 | 12.15±1.98 | 12.37±1.33 | 12.65±1.81 | 12.85±2.19 |

**Arm ratio of individual chromosomes of alfafa.**

| **Chr.** | **XinJiangDaYe** | **ZhongMu No.1** | **GongNong No.1** | **GanNong No.9** | **WL363** | **AErGangJin** | **SaiWo 3** | **HuangHou** | **LeiTing** | **LongMu 803** |
| --- | --- | --- | --- | --- | --- | --- | --- | --- | --- | --- |
| 1 | ^m^1.62±0.09 | ^m^1.20±0.06 | ^m^1.23±0.09 | ^m^1.23±0.13 | ^m^1.21±0.06 | ^m^1.18±0.05 | ^m^1.28±0.23 | ^m^1.18±0.08 | ^m^1.22±0.08 | ^m^1.27±0.10 |
| 2 | ^m^1.17±0.08 | ^m^1.28±0.13 | ^m^1.23±0.10 | ^m^1.23±0.12 | ^m^1.25±0.05 | ^m^1.17±0.06 | ^m^1.20±0.09 | ^m^1.16±0.06 | ^m^1.20±0.08 | ^m^1.17±0.09 |
| 3 | ^m^1.34±0.20 | ^m^1.28±0.19 | ^m^1.29±0.11 | ^m^1.29±0.14 | ^m^1.33±0.13 | ^m^1.41±0.10 | ^m^1.44±0.32 | ^m^1.26±0.11 | ^m^1.49±0.23 | ^m^1.25±0.15 |
| 4 | ^m^1.34±0.14 | ^m^1.38±0.14 | ^m^1.24±0.06 | ^m^1.24±0.11 | ^m^1.30±0.12 | ^m^1.28±0.08 | ^m^1.28±0.17 | ^m^1.30±0.11 | ^m^1.38±0.09 | ^m^1.38±0.15 |
| 5 | ^m^1.22±0.12 | ^m^1.33±0.15 | ^m^1.24±0.10 | ^m^1.24±0.06 | ^m^1.33±0.12 | ^m^1.25±0.11 | ^m^1.22±0.09 | ^m^1.18±0.07 | ^m^1.21±0.06 | ^m^1.35±0.13 |
| 6 | ^m^1.51±0.13 | ^m^1.42±0.04 | ^m^1.34±0.14 | ^m^1.37±0.07 | ^m^1.42±0.25 | ^m^1.45±0.20 | ^m^1.27±0.09 | ^m^1.47±0.21 | ^m^1.40±0.16 | ^m^1.48±0.22 |
| 7 | ^m^1.29±0.15 | ^m^1.36±0.14 | ^m^1.19±0.06 | ^m^1.19±0.12 | ^m^1.39±0.14 | ^m^1.31±0.12 | ^m^1.38±0.18 | ^m^1.25±0.10 | ^m^1.22±0.12 | ^m^1.25±0.12 |
| 8 | ^m^1.14±0.07 | ^m^1.26±0.08 | ^m^1.24±0.14 | ^m^1.24±0.09 | ^m^1.25±0.09 | ^m^1.20±0.08 | ^m^1.24±0.16 | ^m^1.17±0.06 | ^m^1.20±0.08 | ^m^1.14±0.06 |

^m^metacentric
