# Supplementary material for: Oligo-FISH barcode chromosome identification system provides novel insights into the natural chromosome aberrations propensity in the autotetraploid cultivated alfalfa
Source: Hortic Res. 2024 Sep 20;12(1):uhae266. doi: 10.1093/hr/uhae266 (PMC11718389; doi:10.1093/hr/uhae266)
Supplement: Web_Material_uhae266 [file web_material_uhae266.zip › Table S4.docx]

**Table S4. Frequency statistics of chromosomal aberrations in seeds of cultivated alfalfa varieties**

| Alfalfa varieties | Number of seeds with chromosome variations | Total seeds | Chromosome variation type | variant chromosome | Variation frequency (%) |
| --- | --- | --- | --- | --- | --- |
| HuangHou | 1 | 60 | aneuploids (2n+1=33) | chr3 | 1.67 |
|  | 1 |  | aneuploids  (2n+1=33) | chr2 | 1.67 |
| LeiTing | 2 | 59 | aneuploids  (2n+1=33) | chr2 | 3.39 |
|  | 1 |  | chromosome large segment deletions  (2n=32) | chr2 | 1.69 |
| LongMu 803 | 2 | 54 | aneuploids  (2n+1=33) | chr6 | 3.70 |
