# Supplementary material for: Oligo-FISH barcode chromosome identification system provides novel insights into the natural chromosome aberrations propensity in the autotetraploid cultivated alfalfa
Source: Hortic Res. 2024 Sep 20;12(1):uhae266. doi: 10.1093/hr/uhae266 (PMC11718389; doi:10.1093/hr/uhae266)
Supplement: Web_Material_uhae266 [file web_material_uhae266.zip › Table S5.docx]

**Table S5. Statistical analysis of DNA sequence variation among four homologous chromosome copies of each chromosome in Alfalfa**

| Chr | SNPs(Number) | | |  | indels(Number) | | |  | PAVs(Length/bp) | | |
| --- | --- | --- | --- | --- | --- | --- | --- | --- | --- | --- | --- |
|  | chrx.2 | chrx.3 | chrx.4 |  | chrx.2 | chrx.3 | chrx.4 |  | chrx.2 | chrx.3 | chrx.4 |
| chr1.1 | 203238 | 199760 | 197499 |  | 42542 | 41799 | 41583 |  | 419137 | 559926 | 601083 |
| chr2.1 | 175099 | 168552 | 173663 |  | 35986 | 34931 | 35732 |  | 655656 | 394958 | 543592 |
| chr3.1 | 191043 | 181445 | 192717 |  | 37343 | 36609 | 37586 |  | 528866 | 545252 | 466532 |
| chr4.1 | 199882 | 195197 | 194156 |  | 39405 | 38650 | 38079 |  | 617647 | 482214 | 623034 |
| chr5.1 | 184744 | 179640 | 183057 |  | 37423 | 36441 | 36896 |  | 390057 | 509232 | 401691 |
| chr6.1 | 133328 | 120998 | 109618 |  | 20632 | 19254 | 16586 |  | 512603 | 352210 | 401596 |
| chr7.1 | 182761 | 181351 | 185932 |  | 34010 | 33692 | 34335 |  | 532845 | 450165 | 322722 |
| chr8.1 | 163692 | 168226 | 164451 |  | 34528 | 34562 | 34041 |  | 364916 | 340236 | 419876 |
