# Supplementary figures and images for: Oligo-FISH barcode chromosome identification system provides novel insights into the natural chromosome aberrations propensity in the autotetraploid cultivated alfalfa
Source: Hortic Res. 2024 Sep 20;12(1):uhae266. doi: 10.1093/hr/uhae266 (PMC11718389; doi:10.1093/hr/uhae266)

HuangHou  
( $2n+1=33$ )

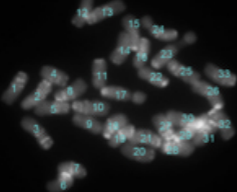

(a)

HuangHou  
( $2n+1=33$ )

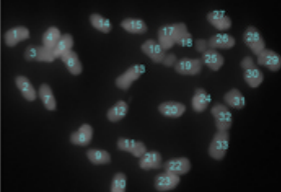

(b)

LeiTing  
( $2n+1=33$ )

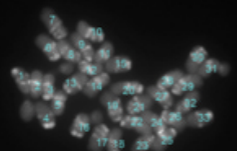

(c)

LongMu 803  
( $2n+1=33$ )

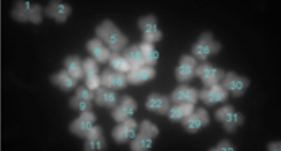

(d)

LeiTing  
( $2n=32$ )

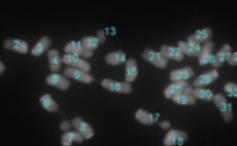

(e)

Supplement: Web_Material_uhae266 [file web_material_uhae266.zip › Fig S1.pdf]
